# Ethical issues in pain and palliation

**DOI:** 10.1097/ACO.0000000000001345

**Published:** 2024-01-30

**Authors:** Marco Cascella, Alessandro Laudani, Giuliana Scarpati, Ornella Piazza

**Affiliations:** a Dipartimento di Medicina, Chirurgia, Odontoiatria ‘Scuola Medica Salernitana’, Università di Salerno; b AOU San Giovanni di Dio e Ruggi d’Aragona, Salerno, Italia

**Keywords:** artificial intelligence, ethics, ICU, opioids, palliative care

## Abstract

**Purpose of review:**

Increased public awareness of ethical issues in pain and palliative care, along with patient advocacy groups, put pressure on healthcare systems and professionals to address these concerns.

Our aim is to review the ethics dilemmas concerning palliative care in ICU, artificial intelligence applications in pain therapy and palliative care, and the opioids epidemics.

**Recent findings:**

In this focus review, we highlighted state of the art papers that were published in the last 18 months, on ethical issues in palliative care within the ICU, artificial intelligence trajectories, and how opioids epidemics has impacted pain management practices (see Visual Abstract).

**Summary:**

Palliative care in the ICU should involve a multidisciplinary team, to mitigate patients suffering and futility. Providing spiritual support in the ICU is an important aspect of holistic patient care too.

Increasingly sophisticated tools for diagnosing and treating pain, as those involving artificial intelligence, might favour disparities in access, cause informed consent problems, and surely, they need prudence and reproducibility.

Pain clinicians worldwide continue to face the ethical dilemma of prescribing opioids for patients with chronic noncancer pain. Balancing the need for effective pain relief with the risk of opioid misuse, addiction, and overdose is a very controversial task.

## INTRODUCTION

Anesthesiologists, especially those involved in the care of very severe or dying patients, are facing daily ethical issues, because of the complex decision-making, which is accomplished with evolving medical technologies, in a context of changing societal values. In the recent medical literature, ethical considerations predominantly revolve around palliative care within ICUs, the ethical implications associated with the integration of cutting-edge technologies, particularly artificial intelligence, and the issues of opioids in pain management and palliative medicine. This comprehensive review will delve into these pivotal ethical concerns (see Supplementary video). 

**Box 1:**
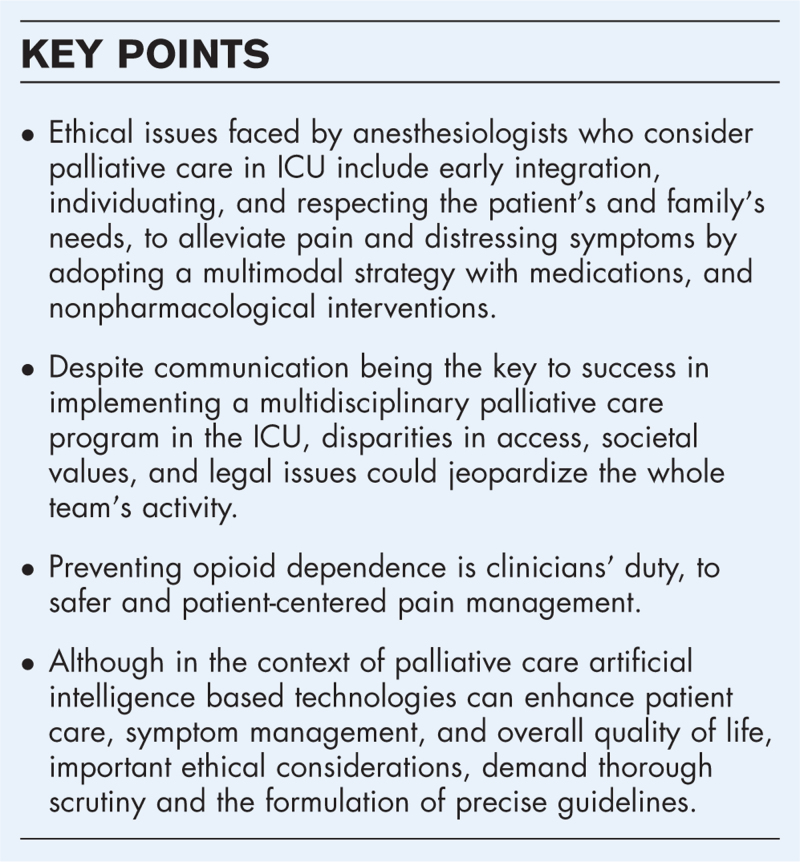
no caption available

## PALLIATIVE CARE IN ICU

Palliative care is providing the best possible care tailored to the patient's individual needs and wishes. Palliative care in ICU is a specialized approach that focuses on providing comfort, symptom management, and emotional support to patients with serious illnesses or life-threatening conditions. Its primary goal is to improve the quality of life for patients and their families, especially when curative treatments may no longer be effective or appropriate.

Nevertheless, there is no agreement on how to recognize patients most likely to benefit of specialist palliative care. Cox *et al.*[1], in a 2022 cohort study, assessed whether palliative care consultations in ICU prompted by clinical characteristics associated with mortality or resource utilization (i.e. worsening organ dysfunction, severe acute neurologic condition, cardiac arrest, advanced cancer, one or more recent ICU admissions, dementia), reflect adequately the real palliative care needs. The Authors [1] concluded that clinical markers of prognosis and resource utilization have serious limitations as palliative care screening tools in ICU, when compared to self-reported palliative care needs. Similarly, Murali *et al.*[2], in a qualitative secondary analysis, by using a dataset containing clinically diverse viewpoints to examine the use of triggers for palliative care consultation in the ICU, concluded new research is needed to find the most appropriate and effective triggers.

The need for palliative care perceived by patients and their families may be measured by the ‘Needs at the End-of-Life Screening Tool’ (NEST) [1], a 13-item questionnaire that assesses needs in several domains of palliative care quality: physical and psychological symptoms, social support, end-of-life care, spiritual and cultural aspects of care. Following on the search for adequate triggers to call in Palliative Care specialists, Luethi *et al.*[3] described a 14 variables prediction model based on a retrospective single-center cohort study, and created the ‘electronic poor outcome screening’ (ePOS) score. The ePOS score is highly sensitive for 6-month mortality, but its goal is to improve the detection of patients with unmet palliative care needs. Ideally, palliative care should be integrated into the care plan early in the course of a serious illness, rather than being introduced as a last resort, and scores as the ePOS, which can be automatically extracted from electronic medical records, may allow early identification of ICU patients at a high risk for poor outcome and potential palliative care needs.

We conceptually support the use of these personalized tools, as they represent an effort towards patient and family-centered outcomes, even if they should be examined thoroughly to establish their actual effectiveness and clinical utility.

Palliative care in ICU can be provided by the patient primary team of intensivists or by palliative care specialists, who have more resources and expertise at their disposal. In our opinion, palliative care in ICU should involve a multidisciplinary team, including physicians, nurses, social workers, chaplains, and other specialists, working together to address the holistic needs of the patient and family. Andersen *et al.*[4^▪^] planned a multicenter efficacy trial recruiting 500 ICU patients over 60 s and their surrogate decision-makers, to compare integrated specialty palliative care versus usual care performed by ICU physicians, by using the modified Patient Perceived Patient-Centeredness of Care scale. The results of the ProPACC trial will be surely interesting and hopefully useful to guide future improvements in supportive and palliative ICU care.

Providing spiritual support in ICU is an important aspect of holistic patient care. The primary goal of spiritual support in the ICU is to provide comfort, solace, and a sense of meaning to the patient during the most challenging and often traumatic time. It should always be provided with respect for the patient autonomy and cultural beliefs, it should be considerate of diverse views, including those who do not identify with any faith. ICU patients and their families often face immense stress, uncertainty, and existential questions Families of ICU patients also require spiritual support but offering them a well tolerated space to express their feelings is very difficult in the ICU setting, since spiritual discussions and practices should be carried out in a private and confidential manner. Prof. Paolo Pelosi [5] in his last lesson wrote: ‘Religion is important among patients and surrogates, but only a minority of medical conferences included any reference to religion or spirituality and/or are attended by spiritual caregivers. This leads to the so-called silence surrounding spirituality and religion in critical care, likely due to opposing environments, one characterised by normal human life, the other by technology and illness.’

After a patient death, palliative care teams should continue to provide support to the family through the grieving process, even if the frantic ICU rhythms dishevel bereavement support.

Palliative care teams should facilitate communication between the ICU team, the patient, and the patient's family to ensure that everyone understands the prognosis, treatment options, and goals of care. Conversations about the patient goals and preferences are essential. These discussions help guide treatment decisions and may involve the development of advance care directives or the identification of a healthcare proxy. Kruser *et al.*[6], addressing the engagement of patients and families in decision making, propose a shift in how clinicians think and communicate about patients, to transitioning to a transparent deliberation about the therapy, which must align with the patient's goals and priorities. ‘As clinicians, we regularly use the word “need’ to think about and describe the condition of patients with acute serious illness……When clinicians, from a position of authority, describe patients with respiratory failure as needing intubation, patients and families presume intubation is what should be done…When a patient is facing a life-threatening illness, instead of saying she ‘needs to be intubated,’ we suggest that clinicians say, ‘Her illness is getting worse. I would like to talk with you about what this means and what to do next’.

## ARTIFICIAL INTELLIGENCE AND ETHICS IN PALLIATIVE CARE

Most artificial intelligence and machine learning research in palliative care is concentrated on predicting mortality and prognosis [7,8] and on the assessment of psychological symptoms and distress [9]. Moreover, artificial intelligence and machine learning techniques have been employed for pain diagnosis in the field of automatic pain assessment that relies on objective methods such as different behaviors and biosignals to assess pain and pain-related features [10]. Utilizing facial expressions and video analyses, researchers developed a binary classifier model for distinguishing between the absence and presence of pain in cancer patients [11^▪^].

Natural language processing (NLP) is a subfield of artificial intelligence and computer science dedicated to human-computer interaction using natural language. These models are adopted for understanding, interpreting, and generating human language [12] and can be a great opportunity for improving care. The applications are manifold and include virtual assistance, Chatbots such as ChatGPT, and ‘sentiment analysis’ to discern emotions or opinions expressed in text or audio. In their recent scoping review on the topic, Sarmet *et al.*[13^▪^] found 32 different NLP software often combined with machine learning or deep learning models, implemented for an array of clinical applications. NLP, for instance, was used to address the issue of serious illness communication [14]. It refers to the process of exchanging information and discussions between healthcare providers and patients (and caregivers) concerning a serious or life-threatening medical condition with discussion on prognosis, advance care planning, and other key aspects of the care. In another NLP-based analysis, Di Martino *et al.*[15] used EHR to detect symptoms of severe or moderate pain, dyspnea, and vomiting/nausea in patients with advanced cancer. From a prospective standpoint, it could be intriguing to apply patient-focused sentiment analysis strategies to assess the extent of the psycho-affective components of pain and key aspects of the care.

Despite the potential advantages of artificial intelligence in palliative care (Table 1), there are numerous ethical considerations to ponder. For example, even with human intervention incorporated into the decision-making process (human in the loop), it remains imperative for the patient to be apprised of the existence of decision-making frameworks grounded in automated processing. Nonetheless, pivotal ethical challenges such as fairness and bias in system design and data training, along with concerns about data privacy, security, and model explainability, underscore the need for robust regulatory frameworks. Effectively tackling these issues demands a collaborative effort involving clinicians, technologists, policymakers, ethicists, and the wider public, collectively shaping the future of artificial intelligence in a responsible and ethical manner [16]. The findings of a recent systematic review revealed a gap between the high-level ethical principles and guidelines proposed by ethicists and the empirical research on the topic [17].

**Table 1:** Examples of recent artificial intelligence applications in palliative care

Aim(s)	AI methods	Data source	Ref.
Prediction of mortality and prognosis	Deep Learning: Three bidirectional LSTM for clinical variables and an ANN for demographic and social history variables.	EHR and clinical and laboratory variables	[8]
Pain Diagnosis	ANN: Binary Classifier (17,1)	Video extracted features (AUs)	[10]
Detection of social distress, spiritual pain, and severe physical and psychological symptoms	ML: LR, RF, LightGBM, SVM	Unstructured data from EHR	[9]
Identification of severe or moderate pain, dyspnea, or vomiting/nausea	NLP (CLARK) plus ML (RF)	EHR	[15]
Serious illness communication analysis	NLP (BERT, Bio+Clinical BERT) plus ML (LG, XGBoost)	EHR (subdomains)	[14]
Prognosis Serious illness communication analysis Precision medicine	ML-based behavioral intervention	RCT including 20 506 patients. Data from EHR.	[18]

AI, Artificial Intelligence; AUs, action units; BERT, bidirectional encoder representations from transformers; CLARK, Clinical Annotation Research Kit; EHR, electronic health records; LightGBM, light Gradient Boosting Machine; LR, logistic regression; LSTM, long short-term memory; ML, machine learning; NLP, natural language processing; RCT, randomized clinical trial; RF, random forest; SVM, support vector machine; XGBoost, extreme gradient boosted trees.

Ethical considerations also arise in how these predictions are communicated, balancing hope and realism in discussions with patients and their families. Moreover, concerns also emerge regarding the appropriate equilibrium between human and artificial intelligence involvement in decision-making, especially in emotionally charged situations. Importantly, the implementation of artificial intelligence technologies can impact patient autonomy, raising questions about informed consent. Therefore, it is crucial to ensure that patients and their families have a comprehensive understanding of the implications of artificial intelligence interventions, covering potential benefits and risks. Artificial intelligence technology should always function as a complementary tool rather than a replacement for a physician. Furthermore, it is crucial to address additional pillars of prudence, embracing a precautionary principle, and reproducibility, ensuring that users can always understand the origins of their answers and guidance [18].

In a randomized clinical trial using artificial intelligence, Manz *et al.*[19] demonstrated that electronic prompts sent to healthcare providers based on artificial intelligence algorithms, which were previously developed for predicting mortality risk, significantly reduced the use of aggressive chemotherapy and other systemic therapies at the end of life. These findings are highly significant, as not properly calibrated interventions are associated with poor quality of life and side effects that can lead to unnecessary hospitalizations [20]. This study offers a commendable example of an artificial intelligence application upholding the principle of nonmaleficence.

## OPIOIDS BEYOND THE ‘PERFECT STORM’

A ‘perfect storm’ has hit the United States and Canada for about two decades. In terms of human lives lost, these countries have higher numbers than the deaths of the first and second worldwide wars added together, resulting from overlapping increases in mortality attributable to three classes of opioids: prescription opioids, heroin, and new illicit opioids of synthetic origin, determining the so-called ‘opioids epidemic’ [21].

The echoes of what happened in the United States and Canada have increasingly influenced the international sphere [22], through continuous media and scientific hammering, which has not spared the world of Hollywood fiction, that is, with the mini-Dopesick series [23].

Although the human effort to eradicate pain and suffering is innate, the principle that any type of pain should always be treated regardless of its nature was only established in the 1980 s.

Unfortunately, the right to pain relief has been often interpreted as a mere reduction in pain intensity scores, and the understanding of that being in pain involves feelings of anger, frustration, hopelessness, and more, is neglected. Such a simplistic approach has involved the practice of prescribing an increasing opioid dosage titrated to achieve just the pain intensity score reduction.

Over time, various institutional interventions have been undertaken to combat the opioid crisis. The CDC Opioid Prescribing Guidelines [24^▪^] were updated in 2022, recommending the importance of flexible, individualized, patient-centered care. This applies both when faced with a new patient and when tapering the dosage of an existing opioid therapy. Furthermore, the CDC guidelines deplore the persistence of barriers to access to evidence-based pain therapy, and underline the importance of sharing decisions by patients and clinicians.

Indeed, the current issue of ethical prescribing involves a transparent communication leading to an individualized treatment plan that considers alternative pain management strategies, and includes opioids only when their benefits outweigh the risks. Moving forward, regular monitoring is essential to assess pain control, functional status, and the development of any adverse effects.

Before prescribing an opioid, it is mandatory for the pain clinician to conduct thorough assessments to identify patients at a higher risk of opioid-related issues. About this, the use of NLP can extract the information of interest from the patient's clinical notes, allowing for structured information that can be used for further analysis. In this way it is possible to obtain information that the patient is reluctant to admit or that is difficult to obtain through routine screening [25].

Machine learning methods for predicting opioid use disorder have been reviewed [26], proving to be reliable, but it is crucial to integrate multiple data types, as well as identify relevant features or variables, to enhance predictive accuracy.

The transition from the era of overprescription to the current policies of underprescription is leading on the one hand to poor treatment for patients with severe chronic pain and on the other to the creation of a new nosological category -- the so-called ‘legacy patients’ -- who often turn to illicit street drug market [27]. As we reported earlier in the text, chronic pain is not limited to the physical domain, but it may trigger a very complex psychological change, and opioids are addictive even when patients no longer take pills for pain, but to feel the euphoric rush of dopamine.

The need for better tools to identify which patients can benefit most from drug therapy while reducing side effects and misuse in particular is invoked by many.

Pharmacogenetics could help choose the most appropriate opioid, if indicated, for an individual.

Ballester *et al.*[28] reviewed the implication of cytochrome P450 2D6 phenotypes on pain relief, analgesic tolerability, and potential opioid misuse.

Genetic differences could at least partially explain the often variable and unpredictable responses to opioids. From a personalized medicine perspective, in the not too distant future, the individual phenotypic profile could be included in each patient's electronic record to guide a safer opioid use.

Avoiding opioid overreliance is a responsibility and moral obligation for pain clinicians. By integrating ethical considerations into the prescribing practices, healthcare providers must contribute to safer and more patient-centered pain management while addressing the societal challenges associated with opioid use. Artificial intelligence can play a significant role, both by identifying patients at risk of addiction and by optimizing the diagnosis and prescribed therapy.

Integrative pain care, as recently emphasized by IASP [29^▪^], should be incorporated into standard clinical practice in order to be offered to the majority of patients.

## CONCLUSION

Ethical issues in pain management and palliative care are timely and relevant because they intersect with numerous factors, including demographic shifts, medical advances, disparities in access, societal values, and legal changes.

Addressing these ethical challenges is crucial for providing compassionate, patient-centered care that respects the autonomy, dignity, and well being of individuals facing serious illness and end-of-life decisions.

## Acknowledgements

*None.*

### Financial support and sponsorship

*None.*

### Conflicts of interest

*The Authors have no conflicts of interest.*

## Supplementary Material

Supplemental Digital Content

## REFERENCES AND RECOMMENDED READING

Papers of particular interest, published within the annual period of review, have been highlighted as:

▪ of special interest
▪▪ of outstanding interest
